# Association of Auditory Steady State Responses with Perception of Temporal Modulations and Speech in Noise

**DOI:** 10.1155/2014/374035

**Published:** 2014-04-14

**Authors:** Venugopal Manju, Kizhakke Kodiyath Gopika, Pitchai Muthu Arivudai Nambi

**Affiliations:** ^1^AWH Special College, Payyanakkal, Kozhikode, Kerala 673 003, India; ^2^Department of Speech & Hearing, School of Allied Health Sciences, Manipal University, Manipal, Karnataka 576 104, India; ^3^Department of Audiology & Speech Language Pathology, Kasturba Medical College, Manipal University, Mangalore, Karnataka 575 001, India

## Abstract

Amplitude modulations in the speech convey important acoustic information for speech perception. Auditory steady state response (ASSR) is thought to be physiological correlate of amplitude modulation perception. Limited research is available exploring association between ASSR and modulation detection ability as well as speech perception. Correlation of modulation detection thresholds (MDT) and speech perception in noise with ASSR was investigated in twofold experiments. 30 normal hearing individuals and 11 normal hearing individuals within age range of 18–24 years participated in experiments 1 and 2, respectively. MDTs were measured using ASSR and behavioral method at 60 Hz, 80 Hz, and 120 Hz modulation frequencies in the first experiment. ASSR threshold was obtained by estimating the minimum modulation depth required to elicit ASSR (ASSR-MDT). There was a positive correlation between behavioral MDT and ASSR-MDT at all modulation frequencies. In the second experiment, ASSR for amplitude modulation (AM) sweeps at four different frequency ranges (30–40 Hz, 40–50 Hz, 50–60 Hz, and 60–70 Hz) was recorded. Speech recognition threshold in noise (SRTn) was estimated using staircase procedure. There was a positive correlation between amplitude of ASSR for AM sweep with frequency range of 30–40 Hz and SRTn. Results of the current study suggest that ASSR provides substantial information about temporal modulation and speech perception.

## 1. Introduction

Speech acoustics have multiple temporal characteristics [1] among which temporal envelope conveys important acoustic cues for speech understanding. Temporal envelope is a slow fluctuation in amplitude which contains much of the information necessary for the identification of syllables, words, and sentences [2–5]. Shannon et al. [6] reported that good speech recognition scores in quiet can be achieved only with envelope cues extracted from as few as four spectral bands. Spectral bands consisting of higher harmonics of speech are amplitude modulated at the rate of fundamental frequency and it is essential to perceive these modulations to perceptually separate target speech and background noise as two different acoustic streams [7, 8].

Temporal envelope of speech can be considered as a complex amplitude modulation, which is a sum of many modulators. Modulation filter banks located in the auditory system split the complex modulations into series of sinusoidal modulations [9]. Modulation sensitive neurons present in upper brainstem constitute this modulation filter bank [10]. Any process that affects the sensitivity of these neurons will lead to poor coding of temporal envelope and may lead to speech perception difficulties. It is necessary to assess the sensitivity to different modulation frequencies independently as different neurons respond to different modulation frequencies. Sensitivity to these modulations can be psychophysically assessed by measuring modulation detection thresholds (MDTs). MDT is obtained by estimating minimum modulation depth required to detect the presence of amplitude modulation in a sound [11–13]. MDTs across different modulation frequencies will reveal the transfer function of the auditory system for modulation frequencies, which is called temporal modulation transfer function (TMTF). TMTF has been widely used to study auditory temporal acuity in normal hearing individuals [11], sensorineural hearing loss individuals [14, 15], cochlear and brainstem implant users [16–21], and developmental dyslexic children [22]. TMTF has helped to characterize the speech perception difficulties in many clinical populations. Kumar et al. [23] obtained modulation detection thresholds (MDTs) at 8, 20, 60, and 200 Hz in noise-exposed individuals and found that MDTs for 200 Hz modulation frequency were significantly related to speech perception in noise. Studies on auditory neuropathy [24–26] and cochlear implants [27, 28] have reported a strong correlation between modulation detection thresholds and speech recognition scores. He et al. [29] used MDT to assess the temporal processing ability of elderly individuals and they attributed poor MDTs to speech understanding difficulties.

All these lines of evidence suggest that TMTF provides valuable information related to speech perception. However, TMTF has to be measured using behavioral paradigms in which the active cooperation of the subject is required. Therefore it becomes challenging while testing the “difficult to test population”. For this reason, there is a need for objective tool for obtaining MDTs. Purcell et al. [30] and Mijares Nodarse et al. [31] studied the usefulness of auditory steady state responses (ASSR) in estimating temporal modulation transfer function. In either of these studies TMTF was estimated by recording ASSR for amplitude modulation sweeps. Stimulus had a fixed modulation depth with modulation frequency swept over a period of time. By applying this technique, these investigators were able to estimate upper cut-off frequency of modulation encoding in the auditory system. However, MDTs at each modulation frequency were not estimated in these studies. Clinically measurement of MDT would be useful in rehabilitation strategies such as envelope expansion techniques which are implemented for the improvement of speech perception in auditory neuropathy patients [32]. By measuring the MDT at different modulation frequencies, modulation sensitivity loss can be estimated. Based on the modulation sensitivity loss, magnitude of enhancement can be determined. Hence, there is a need for an objective tool to estimate MDT. Current study attempts to estimate MDT using ASSR technique.

The sweep techniques used by Purcell et al. [30] and Mijares Nodarse et al. [31] have the potential advantage that they mimic the ecologically relevant stimuli such as speech and music. Both speech and music are a complex auditory stimulus that has prominent amplitude modulations which vary continuously over time. The separation of different amplitude modulation frequencies and tracking of these amplitude modulation changes over time are important for syllabic segmentation, speech recognition [33]. Studies have reported that ASSR for AM sweep could be used to objectively verify the tracking of dynamic modulations by the auditory system [34] and has been proven to be useful in understanding the neurophysiological deficits in dyslexic children [35]. However, there is dearth of information related to association between ASSR for AM sweeps and speech perception. In this experiment we hypothesized that the amplitude tracking ability as assessed by ASSR could be a predictor of speech intelligibility in noise. Hence the second experiment was aimed to test this hypothesis. Assessment of modulation depth perception and AM changes perception provides important information in understanding perceptual deficits in clinical population. Current study evaluates the utility of ASSR as an objective tool to assess the above mentioned perceptual phenomenon.

## 2. Method

### 2.1. Participants

A total of 30 normal hearing individuals (25 females, 5 males) within age range of 18–24 years (mean age = 21 years) participated in experiment 1. 11 normal hearing individuals within age range of 18–24 years participated in experiment 2. All participants were selected using nonrandom sampling technique. The subjects included for the study had audiograms demonstrative of normal hearing thresholds (<15 dBHL pure tone thresholds for octave frequencies from 0.25 to 8 kHz). The participants had a normal middle ear functioning, with “A” type tympanogram and ipsi- and contralateral stapedial reflexes present at 500, 1000, 2000, and 4000 Hz. Subjects with a history of otologic or neurologic diseases or with auditory processing deficits were excluded from the study. All the participants were recruited with an informed consent prior to the conduction of the study. The study protocol was approved by the institutional ethical committee. Data was collected at Department of Audiology, Kasturba Medical College, Mangalore, over duration of March 2012 to February 2013.

### 2.2. Instrumentation

For recording and analyzing ASSR, IHS SmartEP ASSR version 3.92 was used. MATLAB version 7.0 was used to generate and present the signal/stimulus for behavioral estimation of modulation detection thresholds which was routed to GSI-61 clinical audiometer.

### 2.3. Signal Processing

#### 2.3.1. Broad Band Noise with Fixed Modulation Frequency

Broad band white noise was created with a sampling rate of 20000 Hz, which was then filtered between 100 and 7999 Hz using 4th butterworth filter. Broadband noise carrier was refreshed on each presentation. Total duration of the stimulus was one second. Sinusoidal modulators with 60 Hz, 80 Hz, and 120 Hz frequencies were then created with a starting phase of zero degree. Relatively high rates of amplitude modulations were used in the current study and modulation rates in these are necessary for stream segregation [36, 37]. The filtered noise was then amplitude modulated at each modulation frequency with varying modulation depths. Modulation depth ranged from 10% to 100% (10% steps). Stimuli with different modulation depths are loaded into IHS-SmartASSR for the acquisition of ASSR.

#### 2.3.2. Broad Band Noise with Sweeping Modulation Frequency

Sinusoidal sweeping chirps were created with a sampling frequency of 20,000 Hz. These sweeping chirps stimuli were used to amplitude modulate a band limited white noise with the bandwidth of 100–7999 Hz. Stimuli with sweeping amplitude modulations were created for four different frequency ranges including 30–40 Hz, 40–50 Hz, 50–60 Hz, and 60–70 Hz. Stimuli had a total duration of 1 sec which comprised 100 msec unmodulated segment at initial and final position. Middle 800 ms segment was modulated.

#### 2.3.3. Sentences

Tenlists of HINT [38] sentences which were rated familiar by the 6 Indian English speakers who were exposed to English for at least 10 years were taken. These sentences were recorded in digital recording system at 44,100 Hz sampling frequency at 16-bit operating system. These sentences were spoken by an Indian male speaker who is articulatorily proficient and exposed to English for more than 15 years. The four-talker speech babble (2 male and 2 female speakers) with the same long term average spectrum as the target speech was used as the masker.

### 2.4. Procedure

#### 2.4.1. Behavioral Estimation of Modulation Detection Thresholds

The white noise which is amplitude modulated at 60 Hz, 80 Hz, and 120 Hz was used as stimulus. The stimuli were presented using customized program written in MATLAB which were routed through GSI-61 clinical audiometer. Stimuli were presented at 70 dBSPL to the right ear through TDH 39 headphones. Experiments were performed in sound treated audiometric room. Two-down one-up procedure [39] was used for obtaining modulation detection threshold. With this procedure, probability of responses converges at 70.7% point of the psychometric function. Initial modulation depth used was 50% and later modulation depth was adjusted using ratio steps. Modulation depth was decreased by 10% of the previous modulation depth following two consecutive positive responses. Modulation depth was increased by 10% of the previous modulation depth following single negative responses. During each trial, the subject was presented with two noises one after the other in a two-alternative forced choice (2AFC) paradigm. One of these was the noise without any modulation, and the other was the noise which has amplitude modulations. The subject's task was to indicate which of the intervals contained the amplitude modulations. Practice trials were given for all the subjects prior to the actual testing.

#### 2.4.2. Estimation of ASSR Modulation Detection Threshold

Intelligent hearing system (IHS) version 3.92 Smart ASSR was used to record the evoked responses. The subject was seated on a comfortable reclining chair in a sound treated room and was asked to be relaxed throughout the recording session in order to minimize the artifacts. The electrode sites were cleaned using a skin prepping gel and AgCl electrodes were placed using the conventional single channel montage with inverting electrode placed on ipsilateral (right) mastoid, noninverting to vertex and ground on the contralateral (left) mastoid. Absolute electrode impedance and intraelectrode impedance were less than 5000 Ohms and 2000 Ohms, respectively. The white noise which is amplitude modulated at 60 Hz, 80 Hz, and 120 Hz was presented at 70 dBSPL in the right ear through Etymotic ER-3A insert earphones. Responses were elicited at different modulation depths at each modulation frequency. The response is determined automatically by the instrument using frequency weighted averaging method, where “*F*” ratio is calculated between average amplitude of signal and average amplitude of the noise. The modulation depth was decreased in 10-percentage steps. A combination of ascending and descending procedure was used to track the modulation detection threshold. 200 sweeps were presented at 80% modulation depth at 60 Hz, 80 Hz, and 120 Hz. Following this the modulation depth is decreased and responses are recorded at each modulation depth till the level at which there were no responses was observed. The recordings were stopped when the noise floor is <0.74 *μ*V or when 200 sweeps were completed.

#### 2.4.3. ASSR for AM Sweep

The procedure was similar to estimation of ASSR-MDT. However, responses were estimated at fixed modulation depth of 100%. Presentation level was 70 dB SPL. Responses for stimuli with sweeping amplitude modulations at four different frequency ranges including 30–40 Hz, 40–50 Hz, 50–60 Hz, and 60–70 Hz were elicited for the right ear. A total of 200 sweeps were recorded for each stimulus. Each sweep lasted for 1 second. Two recordings were taken at each modulation frequency range.

#### 2.4.4. Speech Recognition Threshold in Noise

The subject's speech recognition threshold in noise (SRTn) was obtained by adjusting the speech-to-noise ratio (SNR). This was achieved by keeping the speech level constant and by reducing the root mean square level of noise. SNR was varied in 2 dB steps using staircase procedure [39]. A total of 6 reversals were administered. Midpoints of last 5 reversals were averaged to obtain SRTn.

## 3. Results

### 3.1. Association between ASSR-MDT and Behavioral MDT

ASSR-MDT was determined by obtaining the minimum modulation depth at which the ASSR could be recorded. This was performed at three different modulation frequencies (i.e., at 60 Hz, 80 Hz, and 120 Hz). The MDTs were determined behaviorally for each subject at these modulation frequencies using transformed up-down procedure. The results from electrophysiological method (ASSR) were compared to behavioral measures of the TMTF. The mean MDTs for 60 Hz, 80 Hz, and 120 Hz modulation frequencies obtained using ASSR and behavioral measures are given in Table 1. Additionally, amplitude changes with the modulation frequency were determined by measuring the ASSR amplitude at fixed modulation depth of 80%.  Table 2 represents amplitude values ASSR at each modulation frequency.

**Figure 1:**
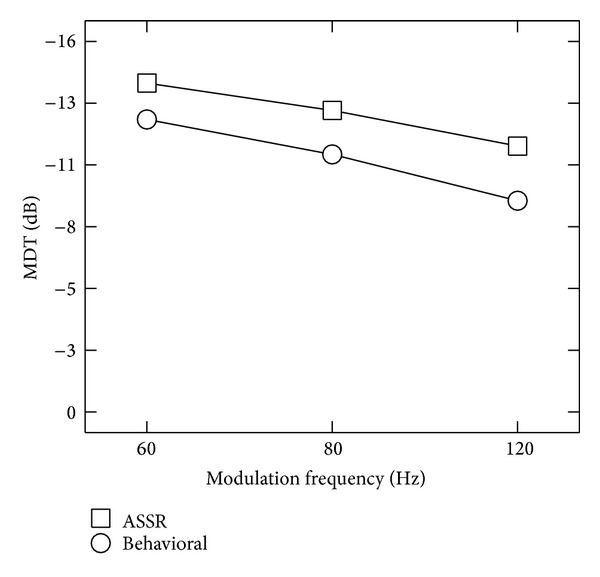
TMTF constructed using mean MDT (dB) obtained using ASSR (rectangles) and behavioral method (circles). (MDT (dB) = 20log⁡(*m*)), where “*m*" is modulation depth in percentage.

It can be seen from Table 1 that there is deterioration in threshold (%) from 19.5 (±9.32) to 26.67 (±10.77) with increase in modulation frequency in both ASSR and behavioral measures. Consistent with previous studies, the ability to identify the amplitude modulation as estimated by MDT became poorer as modulation depth decreased. This holds true for both behavioral measures and ASSR measures. To obtain TMTF, MDTs were plotted against their respective modulation frequencies. Traditionally, TMTF is expressed in dB scale. Hence, MDT in percentage was converted into dB using the formula 20 log⁡_10_⁡(*m*) (where *m* is modulation index). Then the TMTF was constructed using mean MDT which is depicted in Figure 1. It can be observed from the figure that modulation transfer function estimated using ASSR MDT and behavioral MDT is low pass in nature. That is, low modulation frequency has better sensitivity than higher modulation frequencies which is consistent with the literature.

**Figure 2:**
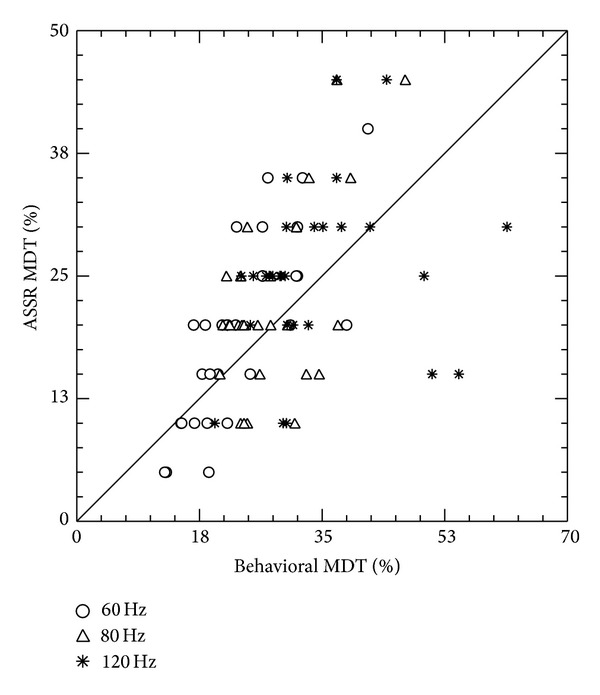
Scatterplot representing association between ASSR MDT and behavioral MDT at 60 Hz, 80 Hz, and 120 Hz modulation frequencies.

Pearson's correlation analysis was used to investigate the association between behavioral and ASSR modulation detection thresholds. The results revealed a significant positive correlation between ASSR modulation detection threshold and behavioral thresholds at 60 Hz (*r* = 0.77; *P* < 0.05), 80 Hz (*r* = 0.58; *P* < 0.05), and 120 Hz (*r* = 0.40; *P* < 0.05). Scatter plots in Figure 2 represent the association between behavioral MDT and ASSR MDT at modulation frequencies 60 Hz, 80 Hz, and 120 Hz.

Predictability of behavioral MDT using ASSR MDT was assessed using linear regression analysis. It was found that for 60 Hz modulation frequency about 60% of variance in behavioral threshold can be attributed to variance observed in ASSR thresholds (*F*(1,28) = 41.78, *P* < 0.05). The linear regression equations are given in Table 3.

### 3.2. Association between ASSR for AM Sweeps and Speech Perception in Noise

**Figure 3:**
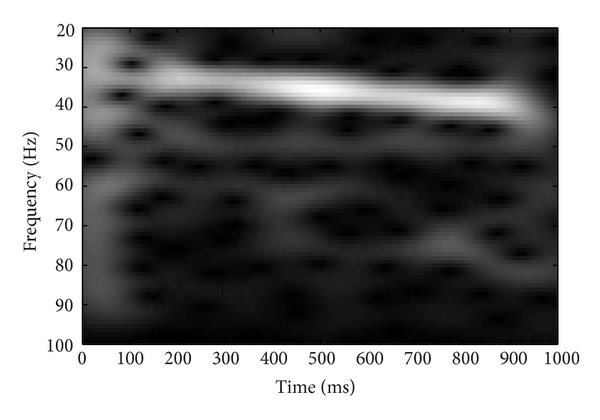
Short time Fourier analysis of the grand averaged response for the frequency range of 30–40 Hz.

**Figure 4:**
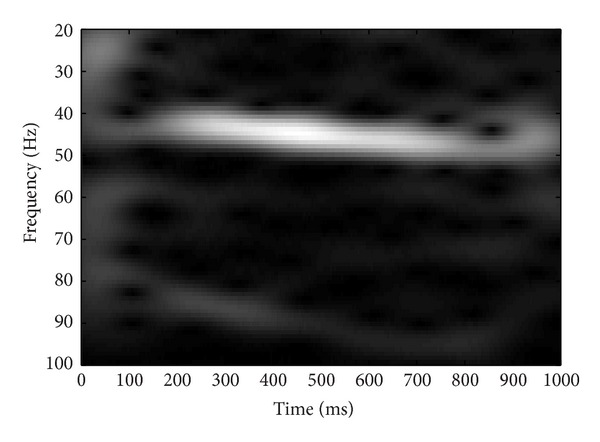
Short time Fourier analysis for the grand averaged response for the frequency range of 40–50 Hz.

**Figure 5:**
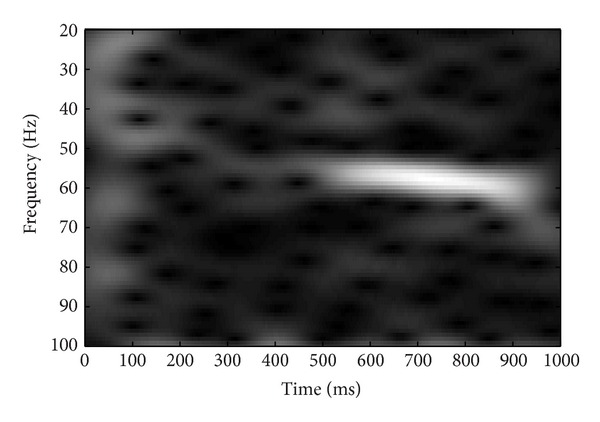
Short time Fourier analysis for the grand averaged response for the frequency range of 50–60 Hz.

**Figure 6:**
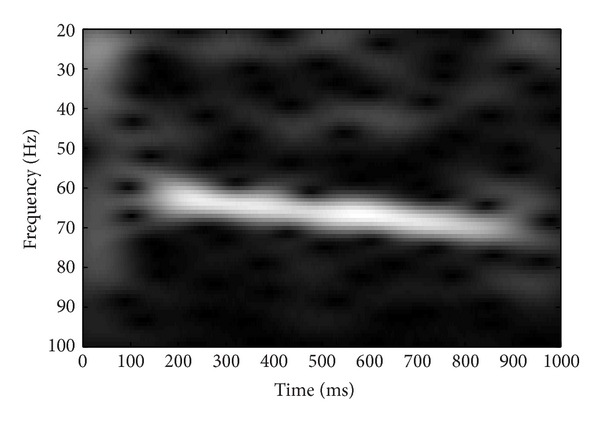
Short time Fourier analysis for the grand averaged response for the frequency range of 60–70 Hz.

Grand average response was derived for each stimulus by summing the response of all subjects. The grand averaged response was subjected to the time frequency analysis. Short time Fourier transform (STFT) was done to analyze the responses in time frequency domain. Analysis was done at 1024-point frequency bin and a hamming window was used to smooth the frequency response. Results of the STFT were represented graphically. STFT analysis confirmed the coding of modulation sweeps at auditory system which provides a physiological evidence for envelope tracking ability of auditory system. Results of the STFT analysis are presented in the form of spectrograms in Figures 3, 4, 5, and 6.

Power analysis in the frequency range of amplitude modulation sweeps was performed for the recorded responses. FFT analysis was performed to identify the evoked responses in the frequency region of the AM sweeps and their corresponding amplitude. RMS amplitudes of the evoked responses in the frequency regions were then calculated for each modulation sweep range. These RMS amplitudes were subjected to further statistical analysis. Mean and standard deviation values for the amplitude of the evoked responses are given in Table 4.

Amplitude of ASSR for AM sweeps was correlated with SRTn to investigate the relationship between speech recognition ability and ASSR. Pearson's correlation analysis was used to assess the possible association between ASSR for AM sweeps and SRTn. The results revealed a significant positive correlation between ASSR for AM sweep and SRTn only at 30–40 Hz range (*r* = 0.61, *P* < 0.05). There was no correlation observed between ASSR and SRTn in other modulation frequencies: 40–50 Hz (*r* = −0.61, *P* > 0.05), 50–60 Hz (*r* = −0.13, *P* > 0.05), 50–60 Hz (*r* = −0.12, *P* > 0.05), and 60–70 Hz (*r* = 0.09, *P* > 0.05).

## 4. Discussion

### 4.1. Association between ASSR-MDT and Behavioral MDT

Based on modulation detection thresholds, the psychophysical temporal modulation rate transfer function (MTF) exhibits a low pass characteristic with MDTs declining with increasing modulation rate. Normal hearing listeners have low threshold for slow modulations and threshold increases as the modulation rate is increased in the TMTF task [11]. Consistent with this, in the present study also the threshold increased when the modulation frequency was increased from 60 Hz to 120 Hz. A similar trend was observed in MDT obtained using ASSR. Even the amplitude of the evoked responses at 80% modulation depth also revealed a low pass modulation transfer function.

**Figure 7:**
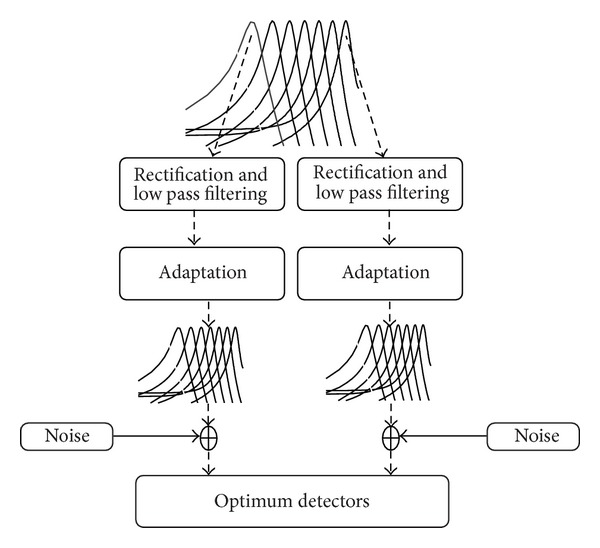
Schematic diagram explaining the mechanism of temporal modulation perception. Mechanism explained here is based on modulation filter bank (MFB) model proposed by Dau et al. [9].

Moderate-to-strong positive correlation was observed between the ASSR and behavioral modulation detection thresholds. Also there was a linear relationship between ASSR-MDT and behavioral MDT. ASSR can be considered as physiological equivalent of subjective modulation perception. Hence, it is reasonable to expect a correlation between these two. Association between ASSR-MDT and behavioral MDT can be explained by relating the ASSR generation to model for amplitude modulation perception (Figure 7) proposed by [9]. This model makes use of the concept called modulation filter bank. According to this model, modulations are extracted from multiple channels at different stages and then they are integrated. A broad band input signal is divided into series of narrow band signals by the peripheral auditory filters. At each filter, the input signal undergoes rectification, compression, and low pass filtering. Output of each auditory filter is fed to the adaptation stage. According to the time constant of adaptation, the envelope is transformed into smooth variations. Then, the transformed envelope is further analyzed by modulation filter banks and later spontaneous neural noise is added to output of each modulation filter. Physiological studies have indicated that probable location of these modulation filters is IC [10]. Neurons in IC are selectively phase locked to different modulation frequencies and further relay them to next stage. This physiological activity is recorded as ASSR using surface electrodes. For the perception of modulation, it undergoes additional stage called optimum detector or also called decision device. With respect to signal detection theory, when an individual is asked to detect the modulations present in the signal, he/she will make a decision based on the sensory information available along with a decision criterion. According to the model at the level between the modulation filter bank and optimal detector the mixing of the internal noise occurs. Introduction of internal noise to the signal reduces the dips which deteriorates the envelope perception. Another factor is that the listener sets a criterion for making a response to maximize the probability of correct responses. If a stringent criterion is adapted by the listener, measured threshold would be high. But, while recording objectively, a decision making process/optimal detector does not play a role.

Mean ASSR thresholds obtained in the current study were slightly smaller than behavioral thresholds. Response bias in the decision making process may be the possible reason for this observed difference. Similarly, trends have been shown in previous attempts to objectively assess the temporal acuity. Werner et al. [40] recorded auditory brainstem responses (ABR) for gaps in noise stimulus. There was a positive correlation between ABR gap detection thresholds (GDT) and psychophysical gap detection thresholds. However, ABR-GDT was smaller than psychophysical GDT. Pratt et al. [41] also have attempted to estimate GDT objectively using long latency responses (LLR). They reported that LLR could code the gap duration of as small as 5 msec and human listeners could identify the gap duration of 5 msec with 60% accuracy. In the current study, 2-down 1-up psychophysical method was used to estimate behavioral threshold, which converges at 70.7% at the psychometric function. If the threshold criterion is set to 70.7% for Pratt et al.'s [41] data, electrophysiological GDT would be smaller than behavioral GDT. Overall results of these studies are in agreement with our finding that objective temporal processing threshold could be better when compared to behavioral thresholds.

### 4.2. Association between ASSR for AM Sweeps and Speech Perception in Noise

STFT analysis confirmed the ability of the auditory system to code sweeping modulation, which provides the physiological evidence for envelope tracking ability of auditory system. There was positive correlation observed between strength of AM sweep coding and speech perception in noise. Response latency, precision of response timing, and response magnitude of specialized IC neurons are the important factors for tracking envelope changes over time [42]. Envelope is tracked by point-by-point sampling and phase locking of auditory neurons at onset of envelope [42]. For a phoneme level sampling, neural oscillations around 40 Hz are important [35]. So, in a connected speech, each phoneme is extracted through a temporal sampling mechanism of these neurons. Under adverse listening condition such as perception speech in noise, the envelope of the speech is smeared. As the background noise fills the temporal dips, the modulation depth reduces thereby smearing envelope [43]. If the auditory neurons are sensitive enough to phase lock the impoverished envelope, good speech recognition can be retained. So, stronger ASSR for AM sweeps can reflect good speech recognition in noise.

## 5. Conclusion

The current study evaluated the utility of ASSR as an objective tool for assessment of temporal modulation perception and speech perception. The first experiment investigated the association between MDT measured using ASSR and behavioral method at 60 Hz, 80 Hz, and 120 Hz. The results of this experiment indicated that there are a strong correlation at 60 Hz and moderate correlation at 80 Hz and at 120 Hz. This suggests that the MDT using ASSR could serve as an objective measure of temporal resolution, which is well correlated with the behavioral measurements. The second experiment explored the association between envelope following response (ASSR) for amplitude modulation sweeps and speech perception in noise. Short time Fourier transform (STFT) analysis confirmed the ability of the auditory system to code sweeping modulation, which provides the physiological evidence for envelope tracking ability of auditory system. The results from ASSR using AM sweep and speech recognition threshold in noise (SRTn) showed a positive correlation between strength of AM sweep coding in 30–40 Hz range and speech perception.

## Figures and Tables

**Table 1:** Mean and standard deviation of ASSR and behavioral MDT.

Modulation frequency (Hz)	ASSR MDT (%)		Behavioral MDT (%)	
	Mean	SD	Mean	SD
60	19.5	9.32	23.35	7.28
80	22.33	9.17	27.81	6.42
120	26.67	10.77	34.98	10.68

Both behavioral and ASSR MDTs are expressed in percentages.

**Table 2:** Mean and standard deviation of ASSR amplitude in dB (20log_10_⁡AMP, Amp in *μ*V) at 80% modulation depth across modulation frequencies.

Modulation frequency (Hz)	Mean (dB)	Standard deviation (dB)
60	19.06	7.76
80	18.3	9.75
120	17.49	7.71

**Table 3:** Regression equations to obtain behavioral MDT from ASSR MDT at each modulation frequency.

60 Hz	80 Hz	120 Hz
*y* = 0.60∗*x* + 11.56	*y* = 0.40∗*x* + 18.78	*y* = 0.0.39∗*x* + 24.64

[*y* =behavioral MDT (%); *x* = ASSR MDT (%)].

**Table 4:** Mean and standard deviation of ASSR amplitude (*μ*v) across different modulation frequency sweeps.

Stimulus	Mean RMS amplitude (*μ*v)	SD (*μ*v)
30–40 Hz sweep	3.90	0.96
40–50 Hz sweep	3.57	0.99
50–60 Hz sweep	3.05	1.31
60–70 Hz sweep	4.09	2.24
